# Circadian photoreception: ageing and the eye’s important role in systemic health

**DOI:** 10.1136/bjo.2008.141747

**Published:** 2008-08-29

**Authors:** P L Turner, M A Mainster

**Affiliations:** University of Kansas School of Medicine, Prairie Village, Kansas, USA

## Abstract

**Aim:**

To analyse how age-related losses in crystalline lens transmittance and pupillary area affect circadian photoreception and compare the circadian performance of phakic and pseudophakic individuals of the same age.

**Methods:**

The spectral sensitivity of circadian photoreception peaks in the blue part of the spectrum at approximately 460 nm. Photosensitive retinal ganglion cells send unconscious information about environmental illumination to non-visual brain centres including the human body’s master biological clock in the suprachiasmatic nuclei. This information permits human physiology to be optimised and aligned with geophysical day–night cycles using neural and hormonal messengers including melatonin. Age-related transmittance spectra of crystalline lenses and photopic pupil diameter are used with the spectral sensitivity of melatonin suppression and the transmittance spectra of intraocular lenses (IOLs) to analyse how ageing and IOL chromophores affect circadian photoreception.

**Results:**

Ageing increases crystalline lens light absorption and decreases pupil area resulting in progressive loss of circadian photoreception. A 10-year-old child has circadian photoreception 10-fold greater than a 95-year-old phakic adult. A 45-year-old adult retains only half the circadian photoreception of early youth. Pseudophakia improves circadian photoreception at all ages, particularly with UV-only blocking IOLs which transmit blue wavelengths optimal for non-visual photoreception.

**Conclusions:**

Non-visual retinal ganglion photoreceptor responses to bright, properly timed light exposures help assure effective circadian photoentrainment and optimal diurnal physiological processes. Circadian photoreception can persist in visually blind individuals if retinal ganglion cell photoreceptors and their suprachiasmatic connections are intact. Retinal illumination decreases with ageing due to pupillary miosis and reduced crystalline lens light transmission especially of short wavelengths. Inadequate environmental light and/or ganglion photoreception can cause circadian disruption, increasing the risk of insomnia, depression, numerous systemic disorders and possibly early mortality. Artificial lighting is dimmer and less blue-weighted than natural daylight, contributing to age-related losses in unconscious circadian photoreception. Optimal intraocular lens design should consider the spectral requirements of both conscious and unconscious retinal photoreception.

Fewer than 1% of retinal ganglion cells are photoreceptive,^1^ but these photoreceptors play a vital role in human physiology and health. Photosensitive retinal ganglion cells (pRGC) were discovered in 2002.^2^ They express the blue-light sensitive photopigment melanopsin^3^ in their cell bodies and elongated dendrites.^4^ Human retinas are spanned by a light sensitive network of roughly 3000 widely dispersed pRGCs.^1^ ^4^ Spectral absorption by melanopsin^2^ and sensitivity of human nocturnal melatonin suppression^5^ ^6^ both peak in the blue portion of the spectrum at 480 and 460 nm, respectively. As shown in fig 1, this short-wavelength sensitivity differs significantly from longer-wavelength peak sensitivities for rod-mediated scotopic (506 nm, green) and cone-mediated photopic (555 nm, green–yellow) vision.^5^ ^7^ ^8^

**Figure 1:**
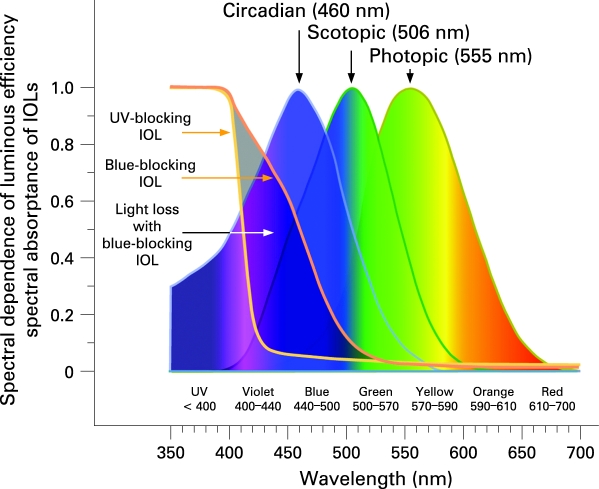
Spectral sensitivity of photopic, scotopic and circadian (melatonin suppression) photoreception.^5^ ^7^ Peak sensitivities of circadian, scotopic and photopic photoreception are 460 nm (blue), 506 nm (green) and 555 nm (green-yellow), respectively. Spectral absorptance is shown for 30D blue blocking (AcrySof SN60AT, Alcon Laboratories, Fort Worth, TX) and UV-only blocking (ClariFlex, Advanced Medical Optics, Santa Ana, CA) intraocular lenses (IOLs).^8^ The area between the two IOL curves is the violet, blue and green light blocked in comparison with a UV-only blocking IOL.

Suprachiasmatic nuclei (SCN) of the anterior hypothalamus serve as the body’s master biological clock.^9^ Ganglion photoreceptors send unconscious, non-visual photic information through the retinohypothalamic tract to the SCN permitting alignment of internal biological with external environmental time. They differ in many ways from the rods and cones that subserve conscious image-based vision.^4^ Ganglion photoreceptors require much more light to respond than cones and have thresholds well above those for photopic vision.^2^ ^9^^–^11 They lack spatial resolution and can adapt to ambient lighting over days^12^ and months.^13^ These properties are well suited to non-directional detection of gross environmental illumination essential for integrated circadian, neuroendocrine and neurobehavioural effects.^4^ Absent or deficient pRGC photoreception cannot be perceived subjectively,^14^ but ensuing circadian disturbances can have significant physiological and psychological consequences.^15^ ^16^

The SCN initiate events timed to allow preparation for impending metabolic, biochemical and physical activities.^15^ Prior to awakening, they activate a morning cortisol surge and trigger changes vital to transitioning from sleep to wakefulness.^14^ Morning exposure to sunlight increases core body temperature,^17^ alerting,^18^ cognition^19^ and brain serotonin levels^20^ which enhance mood and vitality. As the day progresses, peak cognition occurs commensurate with maximal core body temperature. By evening, SCN actively inhibit cortisol secretion for recovery from the morning surge^15^ and initiate pineal secretion of the hormone melatonin which reduces alertness and decreases core body temperature.^14^ As sleep ensues, its slow wave stages and SCN suppression reduce cortisol to a healthy daily nadir as SCN orchestrate a nightly surge of melatonin and other sleep-related hormones.^15^ ^16^ ^21^

Molecular mechanisms controlling self-sustaining SCN clock oscillations have been studied extensively.^22^ Similar mechanisms generating daily rhythms are present in most cells.^23^ Peripheral cell oscillations quickly desynchronise with each other, however, unless constant temporal alignment is provided by the SCN’s neural and hormonal timing signals.^22^ ^23^ Proper SCN functioning is critical for good health due to the numerous functions it coordinates.^15^ ^16^ ^18^ ^21^ ^23^ ^24^ Without robust SCN signals, circadian rhythms of peripheral organs and cells can decouple, producing biochemical disarray and flattened rhythm amplitudes, and increasing risk of disease.^15^ ^25^^–^27

Melatonin produced by the pineal gland is the hormone most closely associated with SCN function.^28^ ^29^ SCN neurons suppress or stimulate melatonin synthesis at appropriate times using a multisynaptic sympathetic pathway.^28^ ^30^ Upregulation of the rate-limiting enzyme in melatonin synthesis (N-acetyltransferase) is directly and immediately suppressed by the SCN in response to light.^10^ ^28^ ^31^ Darkness therefore permits pineal melatonin production during the proper phase of the SCN cycle. Melatonin signals time of day and simultaneously provides potent antioxidant and numerous other beneficial effects.^28^ Experimental nocturnal suppression of melatonin synthesis by light is the widely used surrogate for photic effects on SCN function.^32^

The effectiveness of light exposure for pRGC-mediated biological effects depends on its intensity,^33^ duration,^34^ spectrum^5^ ^6^ and timing relative to the phase of the circadian rhythm.^35^ Internal biological clocks are entrained to external environmental time by timing cues known as zeitgebers.^28^ Daily environmental light is by far the most important zeitgeber in humans,^4^ ^9^ ^10^ ^36^ photoentraining the SCN to light–dark cycles. Suprathreshold early morning light advances, while evening light delays rhythms.^35^

Sunlight has been the primary stimulus for pRGC photoreception throughout human history. Skylight has a dominant wavelength of 477 nm,^37^ similar to peak pRGC sensitivity. Daylight illuminance can exceed 100 000 lux, as shown in fig 2. Contemporary artificial sources rarely provide more than 1% of the brightness of outdoor natural light,^38^ with spectra shifted to longer (redder) wavelengths that are less effective for pRGC photoreception.^39^

**Figure 2:**
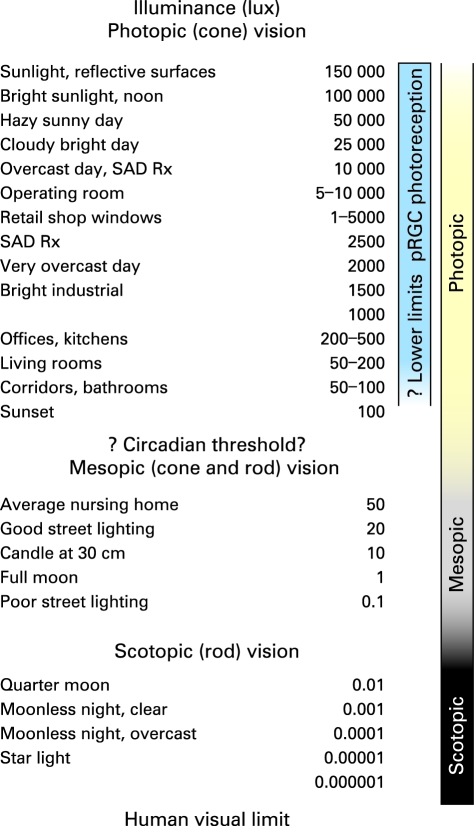
Light levels in contemporary and natural environments^39^ ^54^^–^57 and also in phototherapy for seasonal affective disorder, which is typically 2500 lux for 2 h/day or 10 000 lux for 30 min/day.^44^ Illuminances are given in units of photopic lux. Photopic lux accurately describe the effectiveness of a particular light exposure for overall cone photoreception, which has a peak sensitivity at 555 nm in the green–yellow part of the spectrum (cf, fig 1). A standard circadian lux unit is needed^10^ ^40^ but has not been adopted yet for comparing the effectiveness of different light exposures for circadian photoreception, which has peak sensitivity at 460 nm in the blue part of the spectrum (cf, fig 1).

Brighter, longer, bluer light exposures are most efficient for retinal ganglion mediated effects including melatonin suppression,^5^ ^6^ photoentrainment,^40^ thermoregulation,^17^ improved nocturnal sleep quality,^41^^–^43 heart-rate variability,^17^ treatment of non-seasonal^44^ or seasonal depression,^45^ enhanced mood/well-being,^46^ ^47^ alertness,^17^ ^18^ ^46^ ^48^ cognition,^19^ ^46^ ^49^ reaction time, performance and vigilance.^18^ ^48^ The crystalline lens transmits progressively less visible light and particularly less blue light as it ages.^50^ ^51^ Senescent miosis also progressively reduces retinal illumination.^52^ ^53^ Deficient circadian photoreception results in significant neurobiological morbidity. We therefore examined how ageing and cataract surgery potentially affect the light available for circadian photoreception.

## METHODS

**Figure 3:**
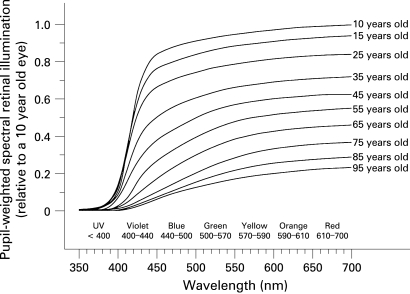
Age-related losses in retinal illumination due to decreasing crystalline lens light transmission and pupil area. Percentage losses per decade are reasonably uniform and most prominent at shorter violet (400–440 nm) and blue (440–500 nm) wavelengths.

**Figure 4:**
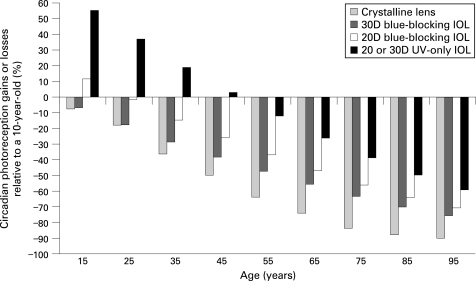
Age-related losses or gains in circadian photoreception relative to a 10-year-old eye for phakic eyes, for 20 and 30D blue-blocking (AcrySof SN60AT, Alcon Laboratories, Fort Worth, TX) and for UV-only blocking intraocular lenses (IOLs) regardless of dioptric power (ClariFlex, Advanced Medical Optics, Santa Ana, CA). Cataract extraction with IOL implantation produces significant gains over phakic eyes, particularly with UV-only blocking IOLs that do not filter out shorter wavelengths vital for non-visual photoreception.

Figure 2 is a compilation of published environmental and therapeutic light levels.^39^ ^44^ ^54^^–^57 Typical indoor and outdoor illuminances were confirmed with standard light meters (Models 403125 and EA30s, Extech Instruments Corporation, Waltham, MA). The age-related decline in retinal illumination in fig 3 was calculated by multiplying human crystalline lens transmittance at different ages^51^ with photopic pupil area for those ages.^53^ Results are presented relative to a 10-year-old eye. Pupil-weighted spectral retinal illumination was multiplied wavelength by wavelength with melatonin suppression sensitivity^5^ ^58^ between 350 and 700 nm to determine how ageing affects circadian photoreception for an isoquantal light source. Resultant areas under the curve for 10 years of age and 15 through 95 years of age represent relative circadian illumination and are presented in table 1. Similar calculations are shown in fig 4 but with the spectral transmittance of 20 and 30-dioptre blue-blocking (AcrySof SN60AT, Alcon Laboratories, Fort Worth, TX) or UV-only blocking (ClariFlex, Advanced Medical Optics, Santa Ana) intraocular lenses^8^ (IOLs) used in addition to that of crystalline lenses (cf, fig 1).

**Table 1:** Relative circadian photoreception vs age*

	10 years	15 years	25 years	35 years	45 years	55 years	65 years	75 years	85 years	95 years
10 years	1.0	0.9	0.8	0.6	0.5	0.4	0.3	0.2	0.1	0.1
15 years	1.1	1.0	0.9	0.7	0.5	0.4	0.3	0.2	0.1	0.1
25 years	1.2	1.1	1.0	0.8	0.6	0.4	0.3	0.2	0.1	0.1
35 years	1.6	1.5	1.3	1.0	0.8	0.6	0.4	0.3	0.2	0.2
45 years	2.0	1.8	1.6	1.3	1.0	0.7	0.5	0.3	0.2	0.2
55 years	2.8	2.5	2.3	1.8	1.4	1.0	0.7	0.5	0.3	0.3
65 years	3.8	3.5	3.2	2.4	1.9	1.4	1.0	0.6	0.5	0.4
75 years	6.1	5.6	5.0	3.9	3.0	2.2	1.6	1.0	0.7	0.6
85 years	8.2	7.6	6.7	5.2	4.1	3.0	2.1	1.4	1.0	0.8
95 years	10.0	9.2	8.2	6.4	5.0	3.6	2.6	1.6	1.2	1.0

*Circadian photoreception declines with ageing due to pupillary miosis and decreased crystalline lens transmission. This table presents circadian performance for an age in the top row relative to that of an age in the left column. For example, a person aged 45 has photoreception roughly half that of a 15-year-old and twice that of a 65-year-old. The table can also be used to estimate the light requirements for an age in the left column relative to that of an age in top row. For example, a person aged 65 needs roughly three times the illuminance of a 25-year-old and half that of a 85-year-old for equivalent circadian photoreception performance.

## RESULTS

Figure 3 shows how losses in crystalline lens transmittance and pupil area due to ageing produce progressive decreases in pupil-weighted spectral retinal illumination. Percentage losses are reasonably uniform with each passing decade. They are most prominent at shorter violet (400–440 nm) and blue (440–500 nm) wavelengths.

Table 1 presents relative effectiveness of circadian photoreception at different ages. By 45 years of age, crystalline lens yellowing and pupillary miosis reduces circadian photoreception to roughly half that of a 10-year-old. People in their eighth and ninth decades retain only 10% of a 10-year-old’s circadian photoreception, so they need 10 times more light for equivalent circadian photoreception under similar illumination, in agreement with Charman’s findings.^58^ Deficits will be underestimates if pRGC populations decline with ageing as do those of non-photoreceptive retinal ganglion cells.^59^ Additional reductions in pRGC photoreception may occur if ocular light transmission is decreased further by factors such as ethnicity, iris pigmentation,^60^ reduced corneal clarity, cataract or sunglass usage.

Figure 4 illustrates age-related losses or gains in circadian photoreception relative to a 10-year-old eye. Cataract extraction with implantation of a UV-only blocking IOL results in significant gains, with performances in older adults comparable with phakic individuals up to four decades younger. People under 50 years of age with UV-only blocking IOLs attain better circadian photoreception than in their youth. UV-only blocking IOLs provide circadian photoreception at any given age roughly 15–20 years younger than blue-blocking IOLs, depending on the latter’s dioptric power.

## DISCUSSION

There is little current information on the susceptibility of retinal ganglion photoreceptors to ocular disease. Retinitis pigmentosa may affect ganglion as well as rod and cone photoreceptors because by 50 years of age, 95% of people with retinitis pigmentosa experience intermittent insomnia, daytime sleepiness and reduced alertness.^61^ ^62^ Glaucoma is associated with ganglion cell losses, but pRGCs were resistant to ocular hypertension in one experimental rodent study.^63^ Cortical blindness would not affect light-mediated pRGC functions so patients should retain normal sleep patterns with appropriate light exposure and potentially benefit from light therapy for coincident depression even though visually blind. Conversely, whiplash injury,^64^ tetraplegia,^30^ autonomic neuropathy or other conditions affecting the retinohypothalamic tract, SCN-pineal connections or intermediate nuclei can impair or abolish specific circadian rhythms.

SCN cycle at fixed, inherited, individually specific periods that typically differ from 24 h and average 24.2 h in humans.^9^ If environmental timing cues are inadequate or absent,^36^ ^65^ SCN cycle daily at their own intrinsic period independent of geophysical day–night cycles. Repetitive cycling without daily resetting is termed free-running.^9^ In free-running, the phase of physiological cycles progressively deviates from and then returns to that of environmental day–night cycles over days or months.

Most totally blind individuals have abnormal or free-running circadian rhythms,^36^ ^66^ but some visually blind individuals retain pRGC photoreception.^67^ Visually blind people without pRGC photoentrainment suffer the additional burden of periodic extreme circadian desynchrony with daytime drowsiness from elevated daytime melatonin levels and night-time insomnia due to circadian alerting.^68^ Their condition is equivalent to a lifetime of recurrent profound jetlag which in itself is disabling.^69^ Blind individuals with intermittent insomnia and daytime napping despite adequate light exposure^36^ should be suspected of free-running. They typically entrain with daily exogenous melatonin, which can improve their quality of life^70^ and possibly reduce otherwise increased early mortality risks.^71^^–^76

Inadequate environmental light exposure can also cause free-running circadian rhythms. People with normal vision in their mid-twenties free-run at room illuminances under 200 lux^77^ or even 80 lux.^78^ Astronauts (37–43 years of age) become free-running at typical space shuttle illuminances below 80 lux, producing circadian disruption, poor sleep quality and neurobehavioural performance decrements.^65^ If 80–200 lux does not prevent free-running with its adverse consequences in 25-year-olds, much higher illuminances would be inadequate for older people with their declining crystalline lens transmittance and pupil area (cf, table 1). For example, 184–460, 256–640, 400–1000 and 536–1340 lux would be inadequate to prevent free-running in 55, 65, 75 and 85-year-old adults, respectively. Residential illuminances are much lower than those needed to prevent free-running in older adults, typically averaging only 100 lux (cf, fig 2).^29^ ^38^ ^57^ This light level is very dim compared with natural outdoor lighting.^39^

Daily light exposures necessary for non-visual photoreception depend on numerous intrinsic^13^ ^60^ ^79^ ^80^ and extrinsic factors.^5^ ^33^^–^35 For example, older women even with dilated pupils are insensitive to blue light exposures sufficient to suppress melatonin significantly in younger women, demonstrating that age-related crystalline lens yellowing reduces circadian photoreception.^81^ As shown in fig 4, cataract surgery provides older adults with more youthful circadian photoreception.

Sunlight’s importance is underscored by seasonal and weather-related neuropsychological disorders that would not occur if indoor lighting were sufficient for all neurobiological needs. Midwinter insomnia affects up to 80% of certain populations at higher latitudes.^82^ Over 90% of people have some mood reduction during sporadically overcast weather or seasonal decreases in daylight length or intensity.^83^^–^85 Seasonal affective disorder (SAD) causes disabling depression, hypersomnolence and weight gain during the fall and winter in approximately 10% of the population.^86^ Non-seasonal depression is also closely associated with reduced light exposure.^87^ ^88^ Reduced sunlight exposure in sighted individuals can cause insomnia, free-running rhythms, extreme flattening of hormonal profiles and cognitive difficulties that are reversible with restoration of adequate sunshine.^89^ ^90^

Environmental illumination is inversely correlated with insomnia^42^ ^91^ and depression,^87^ ^88^ both of which increase with ageing.^92^ Chronic sleep disturbances affect 40–70% of elderly populations.^92^ Indeed, only 12% of 9000 subjects aged 65 or older denied sleep complaints.^93^ Chronic insomnia and depression are closely associated.^93^ ^94^ Up to 30% of older populations have depression,^95^ ^96^ which, like insomnia, frequently goes undiagnosed.^97^ ^98^ Insomnia and depression are significant risk factors for cancer,^99^ diabetes,^100^ cognitive deficiencies,^93^ ^101^ dementia,^102^ cardiovascular disease^95^ and premature mortality.^96^ ^103^ Flattened nocturnal melatonin amplitudes occur with ageing in some^104^ but not all^105^ people probably because of differences in environmental light exposure.^42^ Reduced circadian amplitudes are also associated with higher risks of cancer^106^ and other diseases.^107^ Bright light (⩾2500 lux) particularly from bluer sources such as outdoor daylight can reduce or eliminate insomnia^42^ and depression;^44^ immediately increase brain serotonin,^20^ mood,^47^ alertness, and cognitive function;^17^ ^19^ ^49^ and normalise otherwise decreased circadian hormonal amplitudes including nocturnal melatonin levels that may have been undetectable previously.^42^ ^89^ ^90^

Young adults in industrialised countries typically receive only 20–120 min of daily light exposure exceeding 1000 lux.^42^ ^87^ ^108^ ^109^ Elderly adults’ bright light exposures average only 1/3 to 2/3 that duration.^42^ ^110^ Institutionalised elderly receive less than 10 min per day of light exposure exceeding 1000 lux,^55^ ^111^ with median illuminances as low as 54 lux.^55^ The declining bright light exposure of many older adults combined with their reduced retinal illuminance due to pupillary miosis and crystalline lens yellowing places them at risk for retinal ganglion photoreception deficiency, possibly contributing to age-related insomnia, depression and cognitive decline. Cataract surgery with a UV-only blocking IOL has been shown to decrease the incidence of insomnia and daytime sleepiness.^112^ ^113^

## CONCLUSION

The eye’s critical role in good health has become increasingly evident. Unconscious retinal ganglion photoreceptor responses to bright, properly timed light exposures ensure optimal circadian rhythms, photoentrainment and other neurobiological responses. Inadequate environmental light and/or ganglion photoreception can cause circadian disruption, increasing the risk of insomnia, depression and numerous systemic disorders. Complete blindness involving both conscious vision and unconscious, non-visual photoreception should be differentiated from visual blindness affecting only the conscious perception of light. Visually blind patients should be encouraged to get sufficient light, while completely blind individuals typically benefit from melatonin therapy.

Circadian photoreception decreases with ageing caused by age-related pupillary miosis and reduced crystalline lens transmission, particularly of blue light. Circadian studies should control for subjects’ pupil size and crystalline lens or IOL transmittance. Patient lifestyle education and architectural designs addressing the increased photic needs of older adults are potentially beneficial, as are retinal photocoagulation procedures localised to the outer retina that potentially spare ganglion photoreceptors.^114^ ^115^ Light deficiency, whether due to improper timing, suboptimal spectrum or insufficient intensity, may contribute to medical conditions commonly assumed to be age-related inevitabilities. Unconscious and conscious photoreception should both be considered in IOL design and selection in order to maximise the non-visual as well as visual benefits of cataract surgery.^8^ ^43^

## References

[1] Dacey DM Liao HW Peterson BB Melanopsin-expressing ganglion cells in primate retina signal colour and irradiance and project to the LGN. Nature 2005;433:749–54 15716953 10.1038/nature03387

[2] Berson DM Dunn FA Takao M Phototransduction by retinal ganglion cells that set the circadian clock. Science 2002;295:1070–3 11834835 10.1126/science.1067262

[3] Provencio I Rodriguez IR Jiang G A novel human opsin in the inner retina. J Neurosci 2000;20:600–5 10632589 10.1523/JNEUROSCI.20-02-00600.2000 PMC6772411

[4] Berson DM Strange vision: ganglion cells as circadian photoreceptors. Trends Neurosci 2003;26:314–20 12798601 10.1016/S0166-2236(03)00130-9

[5] Thapan K Arendt J Skene DJ An action spectrum for melatonin suppression: evidence for a novel non-rod, non-cone photoreceptor system in humans. J Physiol 2001;535:261–7 11507175 10.1111/j.1469-7793.2001.t01-1-00261.x PMC2278766

[6] Brainard GC Hanifin JP Greeson JM Action spectrum for melatonin regulation in humans: evidence for a novel circadian photoreceptor. J Neurosci 2001;21:6405–12 11487664 10.1523/JNEUROSCI.21-16-06405.2001 PMC6763155

[7] Wyszecki G Stiles WS Color science: concepts and methods, quantitative data and formulae. New York: Wiley, 1982

[8] Mainster MA Violet and blue light blocking intraocular lenses: photoprotection versus photoreception. Br J Ophthalmol 2006;90:784–92 16714268 10.1136/bjo.2005.086553 PMC1860240

[9] Hannibal J Fahrenkrug J Neuronal input pathways to the brain’s biological clock and their functional significance. Adv Anat Embryol Cell Biol 2006;182:1–71 16566431

[10] Brainard GC Rollag MD Hanifin JP Photic regulation of melatonin in humans: ocular and neural signal transduction. J Biol Rhythms 1997;12:537–46 9406028 10.1177/074873049701200608

[11] Rea MS Figueiro MG Bullough JD A model of phototransduction by the human circadian system. Brain Res Brain Res Rev 2005;50:213–28 16216333 10.1016/j.brainresrev.2005.07.002

[12] Smith KA Schoen MW Czeisler CA Adaptation of human pineal melatonin suppression by recent photic history. J Clin Endocrinol Metab 2004;89:3610–14 15240654 10.1210/jc.2003-032100

[13] Higuchi S Motohashi Y Ishibashi K Less exposure to daily ambient light in winter increases sensitivity of melatonin to light suppression. Chronobiol Int 2007;24:31–43 17364578 10.1080/07420520601139805

[14] Lubkin V Beizai P Sadun AA The eye as metronome of the body. Surv Ophthalmol 2002;47:17–26 11801266 10.1016/s0039-6257(01)00282-x

[15] Buijs RM Scheer FA Kreier F Chapter 20: Organization of circadian functions: interaction with the body. Prog Brain Res 2006;153:341–60 16876585 10.1016/S0079-6123(06)53020-1

[16] Klerman EB Clinical aspects of human circadian rhythms. J Biol Rhythms 2005;20:375–86 16077156 10.1177/0748730405278353

[17] Cajochen C Munch M Kobialka S High sensitivity of human melatonin, alertness, thermoregulation, and heart rate to short wavelength light. J Clin Endocrinol Metab 2005;90:1311–16 15585546 10.1210/jc.2004-0957

[18] Lockley SW Evans EE Scheer FA Short-wavelength sensitivity for the direct effects of light on alertness, vigilance, and the waking electroencephalogram in humans. Sleep 2006;29:161–8 16494083

[19] Vandewalle G Gais S Schabus M Wavelength-dependent modulation of brain responses to a working memory task by daytime light exposure. Cereb Cortex 2007;17:2788–95 17404390 10.1093/cercor/bhm007

[20] Lambert GW Reid C Kaye DM Effect of sunlight and season on serotonin turnover in the brain. Lancet 2002;360:1840–2 12480364 10.1016/s0140-6736(02)11737-5

[21] Czeisler CA Klerman EB Circadian and sleep-dependent regulation of hormone release in humans. Recent Prog Horm Res 1999;**54**:97–130; discussion 130–2. 10548874

[22] Stratmann M Schibler U Properties, entrainment, and physiological functions of mammalian peripheral oscillators. J Biol Rhythms 2006;21:494–506 17107939 10.1177/0748730406293889

[23] Gachon F Nagoshi E Brown SA The mammalian circadian timing system: from gene expression to physiology. Chromosoma 2004;113:103–12 15338234 10.1007/s00412-004-0296-2

[24] Mistlberger RE Circadian regulation of sleep in mammals: role of the suprachiasmatic nucleus. Brain Res Brain Res Rev 2005;49:429–54 16269313 10.1016/j.brainresrev.2005.01.005

[25] Van Someren EJ Riemersma RF Swaab DF Functional plasticity of the circadian timing system in old age: light exposure. Prog Brain Res 2002;138:205–31 12432772 10.1016/S0079-6123(02)38080-4

[26] Hofman MA Swaab DF Living by the clock: the circadian pacemaker in older people. Ageing Res Rev 2006;5:33–51 16126012 10.1016/j.arr.2005.07.001

[27] Hastings MH Reddy AB Maywood ES A clockwork web: circadian timing in brain and periphery, in health and disease. Nat Rev Neurosci 2003;4:649–61 12894240 10.1038/nrn1177

[28] Pandi-Perumal SR Srinivasan V Maestroni GJ Melatonin: Nature’s most versatile biological signal? Febs J 2006;273:2813–38 16817850 10.1111/j.1742-4658.2006.05322.x

[29] St Hilaire MA Gronfier C Zeitzer JM A physiologically based mathematical model of melatonin including ocular light suppression and interactions with the circadian pacemaker. J Pineal Res 2007;43:294–304 17803528 10.1111/j.1600-079X.2007.00477.x PMC2714090

[30] Zeitzer JM Ayas NT Shea SA Absence of detectable melatonin and preservation of cortisol and thyrotropin rhythms in tetraplegia. J Clin Endocrinol Metab 2000;85:2189–96 10852451 10.1210/jcem.85.6.6647

[31] Falcon J Nocturnal melatonin synthesis: how to stop it. Endocrinology 2007;148:1473–4 17369498 10.1210/en.2007-0076

[32] Brainard GC Hanifin JP Rollag MD Human melatonin regulation is not mediated by the three cone photopic visual system. J Clin Endocrinol Metab 2001;86:433–6 11232036 10.1210/jcem.86.1.7277

[33] McIntyre IM Norman TR Burrows GD Human melatonin suppression by light is intensity dependent. J Pineal Res 1989;6:149–56 2915324 10.1111/j.1600-079x.1989.tb00412.x

[34] Czeisler CA The effect of light on the human circadian pacemaker. Ciba Found Symp 1995;**183**:254–90; discussion 290–302. 10.1002/9780470514597.ch14 7656689

[35] Skene DJ Optimization of light and melatonin to phase-shift human circadian rhythms. J Neuroendocrinol 2003;15:438–41 12622847 10.1046/j.1365-2826.2003.01006.x

[36] Skene DJ Lockley SW Thapan K Effects of light on human circadian rhythms. Reprod Nutr Dev 1999;39:295–304 10420432 10.1051/rnd:19990302

[37] Gallagher III FW Beasley WH Gohren CF Green thunderstorms observed. Bull Am Meteorol Soc 1996;77:2889–97

[38] Figueiro MG Rea MS Bullough JD Does architectural lighting contribute to breast cancer? J Carcinog 2006;5:20 16901343 10.1186/1477-3163-5-20 PMC1557490

[39] Thorington L Spectral, irradiance, and temporal aspects of natural and artificial light. Ann N Y Acad Sci 1985;453:28–54 3865588 10.1111/j.1749-6632.1985.tb11796.x

[40] Lockley SW Brainard GC Czeisler CA High sensitivity of the human circadian melatonin rhythm to resetting by short wavelength light. J Clin Endocrinol Metab 2003;88:4502–5 12970330 10.1210/jc.2003-030570

[41] Terman M Lewy AJ Dijk DJ Light treatment for sleep disorders: consensus report. IV. Sleep phase and duration disturbances. J Biol Rhythms 1995;10:135–47 7632987 10.1177/074873049501000206

[42] Mishima K Okawa M Shimizu T Diminished melatonin secretion in the elderly caused by insufficient environmental illumination. J Clin Endocrinol Metab 2001;86:129–34 11231989 10.1210/jcem.86.1.7097

[43] Van Gelder RN Blue light and the circadian clock. Br J Ophthalmol 2004;88:1353 15377569 10.1136/bjo.2004.042861/045120 PMC1772367

[44] Golden RN Gaynes BN Ekstrom RD The efficacy of light therapy in the treatment of mood disorders: a review and meta-analysis of the evidence. Am J Psychiatry 2005;162:656–62 15800134 10.1176/appi.ajp.162.4.656

[45] Glickman G Byrne B Pineda C Light therapy for seasonal affective disorder with blue narrow-band light-emitting diodes (LEDs). Biol Psychiatry 2006;59:502–7 16165105 10.1016/j.biopsych.2005.07.006

[46] Mills PR Tomkins SC Schlangen LJ The effect of high correlated colour temperature office lighting on employee wellbeing and work performance. J Circadian Rhythms 2007;5:2 17217543 10.1186/1740-3391-5-2 PMC1779263

[47] Avery DH Kizer D Bolte MA Bright light therapy of subsyndromal seasonal affective disorder in the workplace: morning vs. afternoon exposure. Acta Psychiatr Scand 2001;103:267–74 11328240 10.1034/j.1600-0447.2001.00078.x

[48] Phipps-Nelson J Redman JR Dijk DJ Daytime exposure to bright light, as compared to dim light, decreases sleepiness and improves psychomotor vigilance performance. Sleep 2003;26:695–700 14572122 10.1093/sleep/26.6.695

[49] Lehrl S Gerstmeyer K Jacob JH Blue light improves cognitive performance. J Neural Transm 2007;14:457–60 10.1007/s00702-006-0621-4 17245536

[50] Boettner EA Wolter JR Transmission of the ocular media. Invest Ophthalmol 1962;1:776–83

[51] Barker FM Brainard GC The direct spectral transmittance of the excised human lens as a function of age (FDA 785345 0090 RA). Washington, DC: US Food and Drug Administration, 1991

[52] Verriest G Influence of age on visual functions in humans. Bull Acad R Med Belg 1971;11:527–78 5153936

[53] Yang Y Thompson K Burns SA Pupil location under mesopic, photopic, and pharmacologically dilated conditions. Invest Ophthalmol Vis Sci 2002;43:2508–12 12091457 PMC2989408

[54] Pears A Strategic study of household energy and greenhouse issues. In: Environment Australia. Canberra: Australian Greenhouse Office, 1998:61–3

[55] Shochat T Martin J Marler M Illumination levels in nursing home patients: effects on sleep and activity rhythms. J Sleep Res 2000;9:373–9 11386204 10.1046/j.1365-2869.2000.00221.x

[56] Lee H-C Introduction to color imaging science. Cambridge: Cambridge University Press, 2005

[57] Knight JA Thompson S Raboud JM Light and exercise and melatonin production in women. Am J Epidemiol 2005;162:1114–22 16207802 10.1093/aje/kwi327

[58] Charman WN Age, lens transmittance, and the possible effects of light on melatonin suppression. Ophthalmic Physiol Opt 2003;23:181–7 12641706 10.1046/j.1475-1313.2003.00105.x

[59] Curcio CA Drucker DN Retinal ganglion cells in Alzheimer’s disease and aging. Ann Neurol 1993;33:248–57 8498808 10.1002/ana.410330305

[60] Higuchi S Motohashi Y Ishibashi K Influence of eye colors of Caucasians and Asians on suppression of melatonin secretion by light. Am J Physiol Regul Integr Comp Physiol 2007 10.1152/ajpregu.00355.2006 17332164

[61] Gordo MA Recio J Sanchez-Barcelo EJ Decreased sleep quality in patients suffering from retinitis pigmentosa. J Sleep Res 2001;10:159–64 11422730 10.1046/j.1365-2869.2001.00251.x

[62] Ionescu D Driver HS Heon E Sleep and daytime sleepiness in retinitis pigmentosa patients. J Sleep Res 2001;10:329–35 11903863 10.1046/j.1365-2869.2001.00271.x

[63] Li RS Chen BY Tay DK Melanopsin-expressing retinal ganglion cells are more injury-resistant in a chronic ocular hypertension model. Invest Ophthalmol Vis Sci 2006;47:2951–8 16799038 10.1167/iovs.05-1295

[64] Smits MG Whiplash injury may deregulate the biological clock. J Neurol Neurosurg Psychiatry 2005;76:1044 16024875 10.1136/jnnp.2004.062034 PMC1739748

[65] Dijk DJ Neri DF Wyatt JK Sleep, performance, circadian rhythms, and light-dark cycles during two space shuttle flights. Am J Physiol Regul Integr Comp Physiol 2001;281:1647–64R 10.1152/ajpregu.2001.281.5.R1647 11641138

[66] Lockley SW Skene DJ Arendt J Relationship between melatonin rhythms and visual loss in the blind. J Clin Endocrinol Metab 1997;82:3763–70 9360538 10.1210/jcem.82.11.4355

[67] Czeisler CA Shanahan TL Klerman EB Suppression of melatonin secretion in some blind patients by exposure to bright light. N Engl J Med 1995;332:6–11 7990870 10.1056/NEJM199501053320102

[68] Lockley SW Skene DJ Tabandeh H Relationship between napping and melatonin in the blind. J Biol Rhythms 1997;12:16–25 9104687 10.1177/074873049701200104

[69] Lewy AJ Emens JS Lefler BJ Melatonin entrains free-running blind people according to a physiological dose–response curve. Chronobiol Int 2005;22:1093–106 16393710 10.1080/07420520500398064

[70] Lewy AJ Bauer VK Hasler BP Capturing the circadian rhythms of free-running blind people with 0.5 mg melatonin. Brain Res 2001;918:96–100 11684046 10.1016/s0006-8993(01)02964-x

[71] Knudtson MD Klein BE Klein R Age-related eye disease, visual impairment, and survival: the Beaver Dam Eye Study. Arch Ophthalmol 2006;124:243–9 16476894 10.1001/archopht.124.2.243

[72] Lee DJ Gomez-Marin O Lam BL Glaucoma and survival: the National Health Interview Survey 1986–1994. Ophthalmology 2003;110:1476–83 12917160 10.1016/S0161-6420(03)00408-1

[73] McCarty CA Nanjan MB Taylor HR Vision impairment predicts 5 year mortality. Br J Ophthalmol 2001;85:322–6 11222339 10.1136/bjo.85.3.322 PMC1723877

[74] Wang JJ Mitchell P Simpson JM Visual impairment, age-related cataract, and mortality. Arch Ophthalmol 2001;119:1186–90 11483087 10.1001/archopht.119.8.1186

[75] West SK Munoz B Istre J Mixed lens opacities and subsequent mortality. Arch Ophthalmol 2000;118:393–7 10721963 10.1001/archopht.118.3.393

[76] Taylor HR McCarty CA Nanjan MB Vision impairment predicts five-year mortality. Trans Am Ophthalmol Soc 2000;**98**:91–6; discussion 96–9. PMC1298215 11190044

[77] Middleton B Stone BM Arendt J Human circadian phase in 12:12 h, 200: <8 lux and 1000: <8 lux light–dark cycles, without scheduled sleep or activity. Neurosci Lett 2002;329:41–4 12161258 10.1016/s0304-3940(02)00574-8

[78] Gronfier C Wright KP Jr Kronauer RE Entrainment of the human circadian pacemaker to longer-than-24-h days. Proc Natl Acad Sci U S A 2007;104:9081–6 17502598 10.1073/pnas.0702835104 PMC1885631

[79] Nathan PJ Burrows GD Norman TR Melatonin sensitivity to dim white light in affective disorders. Neuropsychopharmacology 1999;21:408–13 10457538 10.1016/S0893-133X(99)00018-4

[80] Duffy JF Wright KP Jr Entrainment of the human circadian system by light. J Biol Rhythms 2005;20:326–38 16077152 10.1177/0748730405277983

[81] Herljevic M Middleton B Thapan K Light-induced melatonin suppression: age-related reduction in response to short wavelength light. Exp Gerontol 2005;40:237–42 15763401 10.1016/j.exger.2004.12.001

[82] Nilssen O Lipton R Brenn T Sleeping problems at 78 degrees north: the Svalbard Study. Acta Psychiatr Scand 1997;95:44–8 9051160 10.1111/j.1600-0447.1997.tb00372.x

[83] Spoont MR Depue RA Krauss SS Dimensional measurement of seasonal variation in mood and behavior. Psychiatry Res 1991;39:269–84 1798825 10.1016/0165-1781(91)90093-5

[84] Harmatz MG Well AD Overtree CE Seasonal variation of depression and other moods: a longitudinal approach. J Biol Rhythms 2000;15:344–50 10942266 10.1177/074873000129001350

[85] Dam H Jakobsen K Mellerup E Prevalence of winter depression in Denmark. Acta Psychiatr Scand 1998;97:1–4 9504695 10.1111/j.1600-0447.1998.tb09954.x

[86] Miller AL Epidemiology, etiology, and natural treatment of seasonal affective disorder. Altern Med Rev 2005;10:5–13 15771558

[87] Espiritu RC Kripke DF Ancoli-Israel S Low illumination experienced by San Diego adults: association with atypical depressive symptoms. Biol Psychiatry 1994;35:403–7 8018787 10.1016/0006-3223(94)90007-8

[88] Haynes PL Ancoli-Israel S McQuaid J Illuminating the impact of habitual behaviors in depression. Chronobiol Int 2005;22:279–97 16021844 10.1081/cbi-200053546

[89] Oren DA Giesen HA Wehr TA Restoration of detectable melatonin after entrainment to a 24-hour schedule in a “free-running” man. Psychoneuroendocrinology 1997;22:39–52 9141150 10.1016/s0306-4530(96)00038-8

[90] Bloch KE Brack T Wirz-Justice A Transient short free running circadian rhythm in a case of aneurysm near the suprachiasmatic nuclei. J Neurol Neurosurg Psychiatry 2005;76:1178–80 16024905 10.1136/jnnp.2004.059295 PMC1739733

[91] Hood B Bruck D Kennedy G Determinants of sleep quality in the healthy aged: the role of physical, psychological, circadian and naturalistic light variables. Age Ageing 2004;33:159–65 14960432 10.1093/ageing/afh051

[92] Van Someren EJ Circadian and sleep disturbances in the elderly. Exp Gerontol 2000;35:1229–37 11113604 10.1016/s0531-5565(00)00191-1

[93] Ancoli-Israel S Cooke JR Prevalence and comorbidity of insomnia and effect on functioning in elderly populations. J Am Geriatr Soc 2005;53:264–71S 10.1111/j.1532-5415.2005.53392.x 15982375

[94] Perlis ML Smith LJ Lyness JM Insomnia as a risk factor for onset of depression in the elderly. Behav Sleep Med 2006;4:104–13 16579719 10.1207/s15402010bsm0402_3

[95] Otsuka K Yamanaka G Shinagawa M Chronomic community screening reveals about 31% depression, elevated blood pressure and infradian vascular rhythm alteration. Biomed Pharmacother 2004;**58**(1 Suppl):48–55S 10.1016/s0753-3322(04)80010-6 15754840

[96] Yaffe K Edwards ER Covinsky KE Depressive symptoms and risk of mortality in frail, community-living elderly persons. Am J Geriatr Psychiatry 2003;11:561–7 14506090

[97] Hublin GM Partinen MM The extent and impact of insomnia as a public health problem. J Clin Psychiatry Primary Care Companion 2002;4:8–12

[98] Mecocci P Cherubini A Mariani E Depression in the elderly: new concepts and therapeutic approaches. Aging Clin Exp Res 2004;16:176–89 15462460 10.1007/BF03327382

[99] Connor TJ Leonard BE Depression, stress and immunological activation: the role of cytokines in depressive disorders. Life Sci 1998;62:583–606 9472719 10.1016/s0024-3205(97)00990-9

[100] Spiegel K Knutson K Leproult R Sleep loss: a novel risk factor for insulin resistance and Type 2 diabetes. J Appl Physiol 2005;99:2008–19 16227462 10.1152/japplphysiol.00660.2005

[101] Sheline YI Sanghavi M Mintun MA Depression duration but not age predicts hippocampal volume loss in medically healthy women with recurrent major depression. J Neurosci 1999;19:5034–43 10366636 10.1523/JNEUROSCI.19-12-05034.1999 PMC6782668

[102] Leonard BE Myint A Changes in the immune system in depression and dementia: causal or coincidental effects? Dialogues Clin Neurosci 2006;8:163–74 16889103 10.31887/DCNS.2006.8.2/bleonard PMC3181774

[103] Mallon L Broman JE Hetta J Sleep complaints predict coronary artery disease mortality in males: a 12-year follow-up study of a middle-aged Swedish population. J Intern Med 2002;251:207–16 11886479 10.1046/j.1365-2796.2002.00941.x

[104] Sack RL Lewy AJ Erb DL Human melatonin production decreases with age. J Pineal Res 1986;3:379–88 3783419 10.1111/j.1600-079x.1986.tb00760.x

[105] Zeitzer JM Daniels JE Duffy JF Do plasma melatonin concentrations decline with age? Am J Med 1999;107:432–6 10569297 10.1016/s0002-9343(99)00266-1

[106] Halberg F Cornelissen G Ulmer W Cancer chronomics III. Chronomics for cancer, aging, melatonin and experimental therapeutics researchers. J Exp Ther Oncol 2006;6:73–84 17228527 PMC2742383

[107] Navara KJ Nelson RJ The dark side of light at night: physiological, epidemiological, and ecological consequences. J Pineal Res 2007;43:215–24 17803517 10.1111/j.1600-079X.2007.00473.x

[108] Savides TJ Messin S Senger C Natural light exposure of young adults. Physiol Behav 1986;38:571–4 3823171 10.1016/0031-9384(86)90427-0

[109] Hebert M Dumont M Paquet J Seasonal and diurnal patterns of human illumination under natural conditions. Chronobiol Int 1998;15:59–70 9493715 10.3109/07420529808998670

[110] Campbell SS Kripke DF Gillin JC Exposure to light in healthy elderly subjects and Alzheimer’s patients. Physiol Behav 1988;42:141–4 3368532 10.1016/0031-9384(88)90289-2

[111] Ancoli-Israel S Klauber MR Jones DW Variations in circadian rhythms of activity, sleep, and light exposure related to dementia in nursing-home patients. Sleep 1997;20:18–23 9130329

[112] Asplund R Ejdervik Lindblad B The development of sleep in persons undergoing cataract surgery. Arch Gerontol Geriatr 2002;35:179–87 14764356 10.1016/s0167-4943(02)00022-5

[113] Asplund R Lindblad BE Sleep and sleepiness 1 and 9 months after cataract surgery. Arch Gerontol Geriatr 2004;38:69–75 14599706 10.1016/j.archger.2003.08.001

[114] Bughi S Shaw S Bessman A Laser damage to retinal ganglion cells: the effect on circadian rhythms. J Diabetes Complications 2006;20:184–7 16632239 10.1016/j.jdiacomp.2005.06.006

[115] Mainster MA Decreasing retinal photocoagulation damage: principles and techniques. Semin Ophthalmol 1999;14:200–9 10758220 10.3109/08820539909069538

